# A case report of multiple anesthesia for pediatric surgery: 80 anesthesia applications in a period of 6 years

**DOI:** 10.1186/s12871-018-0639-9

**Published:** 2018-11-20

**Authors:** Sibel Oba, Hacer Şebnem Türk

**Affiliations:** Şişli Hamidiye Etfal Education and Research Hospital, Anesthesiology and Reanimation Department Halaskargazi Cad. Etfal Sok, Istanbul, Şişli Turkey

**Keywords:** Multiple anesthesia, Pediatric, Side effects

## Abstract

**Background:**

The side and adverse effects of anesthesia and its neurotoxicity to children have become major concerns of anesthesiologists in recent years.

Currently, no clinical trials have provided clear evidence indicating the suitable minimum age for a patient’s first anesthetic application, importance of anesthesia duration, number of anesthetic applications or interval between two consecutive anesthesia applications.

A very rare case concerning the side, adverse and neurotoxic effects of multiple anesthesia in a child is presented.

**Case presentation:**

A case of a 9-year-old child who received 80 applications of anesthesia in 6 years because of corrosive esophagitis is presented. The commonly used anesthetic agents were propofol, fentanyl, rocuronium and sevoflurane.

**Conclusion:**

In our case, there were no permanent side or adverse effects due to multiple anesthesia. The minimal psychological and scholastic problems of our case were tied to frequent hospitalization by the pediatric psychiatry consultation.

## Background

The widespread and growing use of anesthesia in infants and young children has made the safety and adverse effects of anesthesia a major concern of anesthesiologists. Pediatric anesthesiologists are increasingly questioned by parents about the risks of anesthetic agents for their children [1–3]. Currently, no clinical trials have provided clear evidence as to the suitable minimum age for the first anesthetic application, importance of anesthesia duration, number of anesthetic applications or the appropriate interval between two consecutive anesthesia applications.

In this case report, as an example of multiple anesthesia, we present a 9-year-old boy who had accidentally ingested household bleach when he was 3 years old and who received 80 applications of general anesthesia in 6 years for treatment of corrosive esophagitis.

## Case presentation

Our case was admitted to Şişli Hamidiye Etfal Education and Research Hospital Pediatric Surgical Department because of caustic ingestion. He had accidentally ingested household bleach from a water bottle when he was 3 years old. At admission, he was vomiting and had erosions and ulcerations of the oral cavity. Upon physical examination, oral nutrition was halted, and intravenous ranitidine and prophylactic antibiotherapy (ceftriaxone) were administered. Laboratory tests, blood values and chest X-ray were normal. At 12 h after caustic ingestion, he received his first anesthesia for endoscopy. Anesthesia was inducted with 2 mg/kg propofol, 1 mcg/kg fentanyl and 0.5 mg/kg rocuronium. He was orotracheally intubated with a 3.5 sized cuffed intubation tube. Anesthesia maintenance was provided with 1–3% sevoflurane in 50% O_2_-N_2_O. At the end of endoscopy and after the return of spontaneous ventilation, 0.04 mg/kg neostigmine and 0.01 mg/kg atropin were injected. The endotracheal tube was removed when the child was fully awake and opened his eyes. For early pain management, paracetamol 10 mg/kg was used.

Deep and circular ulcerations of the esophageal mucosa were deemed Grade IIB according to the classification of ‘corrosive esophagus grading’. After 15 days of liquid diet, the patient received his second anesthesia for an esophageal balloon dilatation operation. During this intervention lasting 30 min, the anesthesia and drug regimens used were the same as in his first operation. The boy recovered from anesthesia uneventfully. Because of his esophageal stricture, an esophageal replacement operation was indicated, but the parents declined this surgical approach. Subsequently, the patient underwent esophageal balloon dilatation operations under general anesthesia each time he complained of symptoms of dysphagia. Gastro-esophageal reflux episodes were treated with lansoprasol tablet 30 mg per day.

Over a 6-year period, with a frequency of approximately once per month, the patient had in total 80 applications of general anesthesia; 70 applications of general anesthesia were performed using propofol, fentanyl, rocuronium and sevoflurane, and 10 used thiopental, fentanyl, rocuronium and sevoflurane. Laboratory monitoring with complete blood count, biochemistry and electrolyte levels were conducted routinely before each operation. The results were normal except for mild leukocytosis observed at several visits. As complications, allergic skin rash was observed eight times at the induction of anesthesia, and bronchospasm occurred fifteen times during the recovery. Steroids and bronchodilator agents were utilized when needed. The most recent esophagoscopy, performed 6 months ago, revealed a normal esophagus. The patient has been asymptomatic with normal laboratory monitoring for the last 6 months. Neurologic and psychiatric examinations were normal. The cognitive function was measured using the Wechsler Intelligence Scale for Children- Revised (WISC-R), which revealed normal level of intelligence with a total score of 97. Child Behavior Checklist 6–18 (CBCL 6–18) was completed by his mother. The questionnaire detected no abnormalities except for minor attention problems. He has successfully attended primary school for the last 2 years. For a short period, he underwent pediatric psychiatry consultations because of attention problems related to frequent hospitalizations, which were managed without any medications.

## Discussion and conclusions

Severe critical events during pediatric anesthesia or anesthetic drug toxicity in pediatric patients have been investigated in many studies [1–4].

In a multicenter observational study, Habre et al. stated that national, regional, and specialist societies must focus on educating anesthesiologists and their teams and implement strategies for quality improvement in pediatric anesthesia [4]. In our case, all anesthesia applications were performed by anesthesiologists experienced in pediatric anesthesia.

Propofol is a potent intravenous hypnotic agent that is widely used in pediatric anesthesia. Propofol has gained popularity for its rapid onset and rapid recovery. However, prolonged propofol administration (> 48 h) at high doses (> 4 mg/kg/h) may cause a rare but frequently fatal complication known as propofol infusion syndrome [5–7].

In an in vitro study, Monni et al. investigated the neurotoxic action of propofol on hypoglossal motoneurons using electrophysiological recordings. They concluded that because of its potential neurotoxic impact on neurodevelopment, propofol should be used with caution in pediatric surgery [8].

In our case, we preferred propofol as the induction agent because of its rapid onset and recovery. None of the 80 anesthesia applications used total intravenous anesthesia, and there was at least a two-week interval between consecutive anesthesia applications.

Fentanyl is a potent opioid receptor agonist with sedative and analgesic effects that is routinely used in pediatric anesthesia. Some clinical trials have shown that fentanyl can prevent emergence agitation under sevoflurane anesthesia in children [9–11].

Sevoflurane is the ideal agent for inhalation induction of anesthesia in children because it is not irritating to the airway. Sevoflurane is also a bronchodilator, and it is not nephrotoxic as long as a fresh gas flow is kept at > 2 l per minute [1]. In their study, Gueli and Lerman concluded that sevoflurane is a well-tolerated induction agent that rarely causes seizures in children [12].

In a previous study, the effect of repeated isoflurane and sevoflurane anesthesia on rat hepatocytes was investigated. The results suggested that the anesthetics do not present considerable hepatotoxicity [13].

In our case, we preferred rocuronium as a muscle relaxant. We think that the antagonism assured by sugammadex represents an additional safety factor for the use of rocuronium, especially in cases of difficult intubation. The use of sugammadex was not required in our case.

Among the drugs used in anesthesia, antibiotics and, rarely, muscle relaxants elicit an allergic reaction. In our case, the clinical manifestation of allergic reaction was a skin rash observed at the induction of anesthesia (in 8 anesthesia applications), most likely caused by rocuronium.

Thiopental, the most commonly used barbiturate in pediatric anesthesia, depresses respiration and induces apnea [1]. Thiopental is not preferred in short cases because it has no analgesic effect and has a late recovery compared with propofol. We were required to use thiopental when propofol was not available in our hospital pharmacy.

Recently, preclinical data along with retrospective clinical studies have suggested that anesthesia could damage brain functions in children by affecting multiple ion channels, receptors and cell signaling processes in the central nervous system. The stage of brain development at the time of exposure to anesthesia and the frequency and cumulative anesthetic doses are some important factors causing neurotoxicity [14–16]. Brambrink et al. reported that a 5-h exposure of infant rhesus macaque brains to isoflurane was sufficient to cause widespread apoptosis of neurons and oligodendrocytes throughout the developing brain [17]. Zou et al. demonstrated that ketamine administration results in a dose-related and exposure-time-dependent increase in neuronal cell death in the developing rat brain [18]. However, the relevance of these laboratory findings to clinical practice remains unclear because it is quite difficult to find or prove the neurological harm that might be caused by anesthesia in clinical studies conducted on children.

Previous clinical studies related to anesthetic neurotoxicity have generally been conducted on children who received only one exposure to general anesthesia. The Pediatric Anesthesia NeuroDevelopment Assessment (PANDA Study) and General Anesthesia compared to Spinal Anesthesia (GAS) study, found strong evidence that exposure for just under an hour to sevoflurane general anesthesia in infancy does not increase the risk of adverse neurodevelopmental outcome at 2 years of age [3, 14, 15].

The Mayo Anesthesia Safety in Kids (MASK) study and the Recognition Memory Study, which assess neurodevelopmental outcomes of anesthesia in young children, are ongoing [19].

Finally, on December 14, 2016, the FDA issued a “Drug Safety Communication” warning that general anesthesia and sedation drugs used in children less than 3 years of age or in pregnant women in their third trimester who undergo anesthesia for more than 3 h or have repeated use of anesthetics “may affect the development of children’s brains” [20].

We believe that in our case report, anesthetic neurotoxicity was not observed because our case received his first anesthesia at 3 years of age and all anesthesia administrations were of short duration (less than 1 hour). The neurologic and psychiatric examinations were normal. The cognitive function of our case, measured using the WISC-R score was normal. WISC-R test determines two aspects of intelligence: verbal and performance intelligence. Our case gained a verbal score of 88 and a performance score of 107 with a total score of 97. CBCL 6–18 was used to assess behavioral and emotional problems. The questionnaire detected only minor attention problems without any emotional impairment. The minimal psychological and scholastic problems of our case were tied to frequent hospitalization by the pediatric psychiatry consultation.

We presented a pediatric case of multiple anesthesia. In this case, there were no permanent side or adverse effects due to multiple anesthesia. To our knowledge, this is the only article in the literature reporting a pediatric case subjected to such a high number of anesthesia applications.

## Abbreviations

CBCL 6–18: Child Behavior Checklist 6–18
FDA: Food and drug administration
GAS: General Anesthesia compared to Spinal Anesthesia
MASK: Mayo Anesthesia Safety in Kids
PANDA: Pediatric Anesthesia NeuroDevelopment Assessment
WISC-R: Wechsler Intelligence Scale for Children- Revised

## References

[1] De Francisci G, Papasidero AE, Spinazzola G, Galante D, Caruselli M, Pedrotti D (2013). Update on complications in pediatric anesthesia. Pediatr Rep.

[2] Nemergut ME, Aganga D, Flick RP (2014). Anesthetic neurotoxicity: what to tell the parent. Paediatr Anaesth.

[3] Sun LS, Li G, Miller TL, Salorio C, Byrne MW, Bellinger DC (2016). Association between a single general anesthesia exposure before age 36 months and neurocognitive outcomes in later childhood. JAMA.

[4] Habre W, Disma N, Virag K, Becke K, Hansen TG, Jöhr M (2017). Incidence of severe critical events in paediatric anaesthesia (APRICOT): a prospective multicentre observational study in 261 hospitals in Europe. Lancet Respir Med.

[5] Kam PC, Cardone D (2007). Propofol infusion syndrome. Anaesthesia.

[6] Orsini J, Nadkarni A, Chen J, Cohen N (2009). Propofol infusion syndrome: case report and literature review. Am J Health Syst Pharm.

[7] Fodale V, La Monaca E (2008). Propofol infusion syndrome: an overview of a perplexing disease. Drug Saf.

[8] Monni L, Ghezzi F, Corsini S, Nistri A (2017). Neurotoxicity of propofol on rat hypoglossal motoneurons in vitro. Neurosci Lett.

[9] Niesters M, Overdyk F, Smith T, Aarts L, Dahan A (2013). Opioid-induced respiratory depression in paediatrics: a review of case reports. Br J Anaesth.

[10] Shi F, Xiao Y, Xiong W, Zhou Q, Yang P, Huang X (2015). Effects of fentanyl on emergence agitation in children under sevoflurane anesthesia: meta-analysis of randomized controlled trials. PLoS One.

[11] Tan Y, Shi Y, Ding H, Kong X, Zhou H, Tian J (2016). μ-Opioid agonists for preventing emergence agitation under sevoflurane anesthesia in children: a meta-analysis of randomized controlled trials. Paediatr Anaesth.

[12] Gueli SL, Lerman J (2013). Controversies in pediatric anesthesia: sevoflurane and fluid management. Curr Opin Anaesthesiol.

[13] Ruxanda F, Gal AF, Raţiu C, Miclăuş V, Rus V, Oana LI (2016). Comparative immunohistochemical assessment of the effect of repetitive anesthesia with isoflurane and sevoflurane on rat liver. Braz J Anesthesiol.

[14] Davidson AJ, Disma N, de Graaff JC, Withington DE, Dorris L, Bell G (2016). Neurodevelopmental outcome at 2 years of age after general anaesthesia and awake-regional anaesthesia in infancy (GAS): an international multicentre, randomised controlled trial. Lancet.

[15] Wilder RT, Flick RP, Sprung J, Katusic SK, Barbaresi WJ, Mickelson C (2009). Early exposure to anesthesia and learning disabilities in a population based birth cohort. Anesthesiology.

[16] Jevtovic-Todorovic V, Absalom AR, Blomgren K, Brambrink A, Crosby G, Culley DJ (2013). Anaesthetic neurotoxicity and neuroplasticity: an expert group report and statement based on the BJA Salzburg seminar. Br J Anaesth.

[17] Brambrink AM, Back SA, Riddle A, Gong X, Moravec MD, Dissen GA (2012). Isoflurane induced apoptosis of oligodendrocytes in the neonatal primate brain. Ann Neurol.

[18] Zou X, Patterson TA, Sadovova N, Twaddle NC, Doerge DR, Zhang X (2009). Potential neurotoxicity of ketamine in the developing rat brain. Toxicol Sci.

[19] Pinyavat T, Warner DO, Flick RP (2016). Summary of the update session on clinical neurotoxicity studies. J Neurosurg Anesthesiol.

[20] Andropoulus DB, Greene MF (2017). Anesthesia and developing brains-implications of the FDA warning. N Engl J Med.

